# Impact of Long-Term Care Insurance on health out-of-pocket expenditure ratios for older adults

**DOI:** 10.3389/fpubh.2025.1616761

**Published:** 2025-07-15

**Authors:** Zhe Liu, Ruolin Shi, Lexue Jiang, Fengwen Tang, Zenghui Qiu, Lan Yao

**Affiliations:** ^1^School of Medicine and Health Management, Tongji Medical College, Hugzhong University of Science and Technology, Wuhan, China; ^2^School of Foreign Languages, Huazhong University of Science and Technology, Wuhan, China; ^3^School of Nursing, Tongji Medical College of Huazhong University of Science and Technology, Wuhan, China; ^4^School of Sociology, Nankai University, Tianjin, China

**Keywords:** long-term care, Long-Term Care Insurance, health expenditure, out-of-pocket expenditure, China

## Abstract

**Background:**

China's rapidly aging population has intensified the demand for long-term care (LTC), resulting in higher out-of-pocket expenditure (OOPE) ratios and financial strain among older adults. To mitigate these burdens, Long-Term Care Insurance (LTCI) has been piloted across selected cities. However, its effectiveness in reducing financial burden—particularly OOPE ratios—remains insufficiently evaluated. This study assesses the causal impact of LTCI on OOPE ratios and explores subgroup heterogeneity.

**Methods:**

We used panel data from the China Health and Retirement Longitudinal Study (CHARLS, 2015–2020), applying difference-in-differences (DID), dynamic DID, and propensity score matching DID (PSM-DID) approaches. The treatment group consisted of older adults living in 12 LTCI pilot cities, while controls were drawn from non-pilot areas. We adjusted for socio-demographic and health covariates and conducted robustness and parallel trend tests.

**Results:**

DID results show that LTCI significantly reduced OOPE ratios (*coefficient* = −0.035, *p* < 0.01), with dynamic DID confirming a 5.6% reduction in the post-treatment period. PSM-DID estimates remained consistent (*coefficient* = −0.019, *p* < 0.05). Subgroup analysis revealed stronger effects among relatively younger individuals within the older adult population (70–79), rural residents, and individuals with lower education or chronic conditions. In contrast, minimal impact was observed among those aged ≥80 and highly educated individuals. Notably, OOPE ratios continued to increase over time, indicating that inflation and systemic cost pressures may offset policy gains.

**Conclusions:**

While LTCI has demonstrable short-term benefits in reducing OOPE ratios and improving equity, long-term sustainability remains at risk due to persistent cost escalation. Targeted policy design and enhanced integration with broader health financing mechanisms are needed to strengthen its long-term impact.

## 1 Introduction

### 1.1 Background

As population aging accelerates globally, the demand for sustainable LTC systems has become a pressing concern for health policymakers. China, home to the world's largest older adults population, is experiencing a demographic transition of unprecedented scale and speed. Between 2000 and 2023, the proportion of individuals aged 65 and over increased from 88.27 million (7%) to 216.76 million (15.4%). According to projections by Li et al. (1), China will enter a “super-aged” phase by 2030, with the number of disabled and semi-disabled older adults reaching 69.53 million (17.44%) and possibly increasing to 120.6 million (22%) by 2050.

This demographic transformation has led to a rapidly growing demand for LTC services, particularly among older adults with functional impairments or cognitive decline who require support with daily living activities (2). In the face of diminishing family caregiving capacity and rising care needs, financial pressures on households have intensified. The OOPE ratio, defined as the share of healthcare costs paid directly by individuals, is a critical indicator of financial burden. High OOPE ratios may reduce access to necessary care, exacerbate socioeconomic inequities, and increase the risk of catastrophic health spending (3, 4).

To mitigate these risks, LTCI has emerged globally as a financing mechanism to redistribute care costs and enhance access to formal services. International experiences—such as Germany's mandatory social LTCI (1995) and Japan's universal public LTCI (2000)—demonstrate diverse approaches to institutional or home-based care coverage and eligibility criteria, while sharing a common goal of financial protection (5). These systems generally provide benefits for individuals assessed to have moderate-to-severe disabilities, including cash or in-kind services delivered through certified providers. However, existing evaluations often focus on utilization patterns or total spending, overlooking ratio-based metrics such as the OOPE burden. China began piloting its LTCI system in 2016 across 15 cities in response to mounting care challenges. This system was primarily targeted at functionally dependent older adults, offering benefits in the form of institutional and home-based care services. Eligibility is typically determined through standardized disability assessments, with coverage initially limited to enrollees of the Urban Employee Basic Medical Insurance (UEBMI), although some cities have gradually extended access to Urban-Rural Resident Basic Medical Insurance (URRBMI) participants. By 2023, the LTCI program had expanded to 49 cities, covering over 180 million people, with significant local variation in benefit generosity, service delivery, and enrollment thresholds (6, 7).

A growing body of empirical studies has assessed the early impacts of China's LTCI pilot. For example, Li and Zhang (8) identified reductions in hospitalization-related OOPE, while Lei et al. (9) found a 23.5% decrease in total OOPE per additional year of LTCI participation. Nonetheless, existing research tends to focus on aggregated expenditure outcomes and rarely accounts for subgroup heterogeneity or relative burden measures. Moreover, little attention has been paid to whether LTCI achieves its stated goal of financial protection for the most vulnerable older populations.

To address these gaps, this study offers a comprehensive evaluation of LTCI's impact on the OOPE ratio among older adults in China. Using nationally representative panel data from CHARLS (2015–2020), we employ a multi-stage quasi-experimental design. This includes a standard difference-in-differences (DID) model to estimate average treatment effects, an event-study-based dynamic DID to examine policy effect trajectories, and a PSM-DID approach to mitigate selection bias. Robustness checks—including placebo tests and fixed effects panel models—further enhance the credibility of our findings. We also conduct detailed heterogeneity analyses by age, education, cognitive ability, and health status.

This study makes three key contributions. First, it introduces ratio-based indicators that offer a policy-relevant measure of financial burden. Second, it integrates multiple quasi-experimental methods in a large-scale natural policy experiment. Third, it reveals substantial subgroup differences, highlighting LTCI's redistributive potential as well as equity and sustainability challenges. These findings provide valuable insights for optimizing China's LTCI system and informing global discussions on effective aging policies.

### 1.2 Research hypothesis

Drawing on the framework of financial protection in aging societies, this study proposes two key assumptions to facilitate causal identification and interpret the impact of LTCI on OOPE ratios among older adults:

**H1: All individuals aged 65 and above residing in LTCI pilot cities are assumed to be enrolled in the LTCI program**.

Mechanism: The LTCI scheme in China primarily targets functionally dependent older adults and is administered through the UEBMI and URRBMI. These two schemes together constitute China's basic medical insurance system, whose coverage reportedly exceeds 95% of the national population according to official statistics. Based on this extensive coverage, it is reasonable to assume that nearly all older adults in pilot cities are effectively included in the LTCI system, either directly or by eligibility. This assumption facilitates clear treatment assignment in a quasi-experimental framework and aligns with established practice in policy evaluation studies.

**H2: All long-term care expenses incurred by covered individuals fall within the reimbursement scope of LTCI**.

Mechanism: While in practice LTCI reimbursement rules vary across pilot cities—with local discretion over benefit packages, eligible services, and reimbursement ceilings—this assumption provides a standardized impact pathway. It allows us to interpret changes in OOPE ratios as being attributable to LTCI coverage rather than differences in policy generosity or service inclusion. By simplifying the benefit structure, we ensure comparability across cities and enhance internal validity in estimating net financial protection effects.

These assumptions underpin the empirical strategy described in Section 2.2, where a DID model is used to estimate the average treatment effect of LTCI coverage. They also inform the heterogeneity analyses in Section 3.5, which explore variation in policy impact across age, health, and socioeconomic strata.

## 2 Data and methodology

### 2.1 Sample and data sources

The data used in this study were sourced from three rounds of the CHARLS conducted in 2015, 2018, and 2020 (10). CHARLS is a nationwide, large-scale, multidisciplinary social survey designed to collect extensive micro data on health, work, and retirement issues. The baseline survey was conducted in 2011, with follow-up surveys conducted at 2- to 3-year intervals, and the most recent data updated through 2020. The survey is conducted in July and August of each survey year using a stratified random sampling methodology, covering a nationally representative sample of 150 counties and 450 communities (villages) across 28 provinces. It includes data on basic personal information, household structure, health status, health service utilization, health insurance, and retirement and pensions. Detailed information on CHARLS data is available on its official website (2015 http://charls.pku.edu.cn/en).

This study focuses on older adults aged 65 and above, using data from the 2015, 2018, and 2020 waves of CHARLS to evaluate the impact of China's LTCI pilot policy through a double-DID approach. Only the first batch of 15 pilot cities launched in 2016 was included, as the second batch, announced in September 2020, had no practical impact on the survey data. Due to data limitations, three cities (Changchun, Nantong, and Shihezi) were excluded, leaving 12 pilot cities for analysis. The treatment group consists of individuals aged 65 and above in these pilot cities who were enrolled in basic medical insurance, while the control group includes similarly aged individuals in non-pilot areas. After excluding cases with over 50% missing data and winsorizing continuous variables at the 1st and 99th percentiles, the final analytic sample included 18,973 individuals from an initial pool of 20,808.

### 2.2 Baseline difference-in-differences (DID) regression model

To assess the relationship between LTCI and OOPE ratios among older persons, this study employs the DID method. As a quasi-experimental approach, DID is based on a counterfactual framework that divides individuals affected by a policy shock into treatment and control groups, comparing changes in the dependent variable under conditions where the policy is implemented or not. This method helps mitigate external effects and selection bias, making it widely applied in econometrics and sociology.

The benchmark regression model for this study is specified as follows:


(1) Yi,t=α+βTreatiP*ostt + θXi + μi + γt + ϵi,t

Where *i* and *t* denote individuals and time, respectively; *Y*_*i, t*_ is the explained variable, representing the OOPE ratio for older adults; *Treat*_*i*_ is a treatment group dummy variable that equals 1 if an individual is covered by LTCI and 0 otherwise; *iPost*_*t*_ is a policy pilot dummy variable that takes the value of 1 in the post-pilot period and 0 in the pre-pilot period. The interaction term *Treat_i_* ^*^Post_t_ indicates whether an individual is covered by LTCI and serves as the explanatory variable at the time *t*. *X*_*i*_ represents a set of control variables, μ_*i*_ denotes individual fixed effects, γ_*t*_ represents time-fixed effects, and ϵ_*i, t*_ is a random error term.

### 2.3 Dynamic difference-in-differences specification

To examine the temporal evolution of policy effects and formally test the validity of the parallel trend assumption, we adopt a dynamic difference-in-differences (DID) model based on an event-study specification. Following the standardized panel event study framework proposed by Clarke and Tapia-Schythe (11), this approach enables transparent estimation of dynamic treatment effects in settings with staggered policy implementation. In this framework, an event time variable was constructed to indicate the relative position of each survey year with respect to the LTCI policy implementation year (2016) (11). Specifically, since the LTCI policy was launched in 2016, we define the policy implementation year as *event*_*time* = 0. Given the CHARLS survey waves, 2015 was coded as *event*_*time* = −1, 2018 as *event*_*time* = +2, and 2020 as *event*_*time* = +4. To facilitate estimation, this variable was shifted to create *event*_*time*_*shift*, ranging from one to five, which was then interacted with the treatment group indicator (dealgroup) to capture year-specific treatment effects.

The dynamic DID model takes the following form:


(2) $$\begin{array}{r} Y_{it}=\underset{k\neq 5}{\sum }\beta_{k}\left({event\text{\_}time\text{\_}shift}_{it}=k\times Treatment_{i}\right)\\ +X_{it}\gamma +\mu_{i}+\lambda_{t}+\varepsilon_{it} \end{array}$$

where *i* and *t* index individuals and time, respectively; *Y*_*it*_ represents the natural logarithm of the OOPE ratio for individual *i* at time *t*; *Treatment* is a binary indicator equal to 1 for respondents residing in LTCI pilot cities and 0 otherwise; *X*_*it*_ denotes a set of time-varying individual-level covariates (e.g., demographic characteristics, self-rated health, cognitive function, insurance participation); μ_*i*_ and λ_*t*_ denote individual and time fixed effects, respectively; and ε_*it*_ is the idiosyncratic error term.

In this specification, the omitted category corresponds to the policy implementation year (2016). Since CHARLS does not contain data for 2016, we define *event*_*time* = 0 as 2016 and use subsequent waves (2018 and 2020) to evaluate policy dynamics. The omitted category is thus set to *event*_*time*_*shift* = 3, which serves as the reference point. The coefficients β_*k*_ capture the dynamic policy effects relative to this base period. The 2015 wave (*event*_*time* = −1) is used to assess the plausibility of the parallel trend assumption, while 2018 (*event*_*time* = +2) and 2020 (*event*_*time* = +4) reflect the magnitude and trajectory of the LTCI policy's impact on OOPE ratios over time.

This approach allows for a more nuanced understanding of the temporal dynamics of LTCI policy implementation, and provides additional robustness to the causal interpretation of the estimated treatment effects.

### 2.4 Parallel trend test

The parallel trend test is a fundamental prerequisite for the validity of the Dynamic Dynamic Difference-in-Differences (DID) model. Its core proposition states that before policy intervention, the dependent variables of the treatment and control groups should follow identical time trends. If this assumption fails to hold, the DID estimates may suffer from bias.

To assess the dynamic effects of the policy intervention and examine the validity of the parallel trends assumption, we employ an event-study specification (dynamic difference-in-differences model), which allows treatment effects to vary flexibly across time periods relative to the policy implementation. The parallel trend model takes the following form:


(3) $$\begin{array}{l} Y_{it}=\alpha +\underset{k\neq -1}{\sum }\beta_{k}D_{it}^{\left(k\right)}+\gamma X_{it}+\mu_{i}+\lambda_{t}+\varepsilon_{it} \end{array}$$

where *Y*_*it*_ denotes the outcome variable for individual *i* at time *t* (e.g., health expenditure share, healthcare utilization rate, etc.), α is the constant term, $D_{it}^{\left(k\right)}$ is a set of event-time dummy variables (taking the value of 1 if individual *i* is observed in period *k* before or after the intervention, and 0 otherwise, with the period immediately before the intervention, *k* = −1, serving as the baseline), β_*k*_ captures the dynamic treatment effect at event time *k* relative to the baseline period, *X*_*it*_ is a vector of time-varying covariates (including control variables such as demographic characteristics and health status), γ is the *coefficient* vector for the control variables, μ_*i*_ and λ_*t*_ represent individual fixed effects and time fixed effects (to control for time-invariant heterogeneity and common time shocks, respectively), and ε_*it*_ is the idiosyncratic error term. This model systematically examines the dynamic evolution of policy effects through the series of β_*k*_ coefficients.

A critical identification assumption underlying the DID framework is the parallel trends assumption. This assumption requires that, in the absence of the policy intervention, the treated and control groups would have followed similar trends in the outcome variable over time.

Formally, the parallel trends assumption can be stated as:


(4) $$\begin{array}{l} H_{3}:\beta_{k}=o\;for\;all\;k<0,\;k\neq 1 \end{array}$$

That is, before the intervention, there should be no significant differences in outcome trends between the treated and control groups relative to the reference period. Failure to satisfy this condition would suggest that the estimated post-treatment effects may be confounded by pre-existing differential trends. To empirically evaluate this assumption, we conduct joint significance tests on the pre-treatment event-time coefficients (*k* < 0).

### 2.5 Variables and their operationalization

#### 2.5.1 Dependent variable: proportion of OOPE for health of older persons (OOPE ratio)

The primary dependent variable in this study is the OOPE ratio, defined as (total LTC costs—total reimbursements)/total LTC costs. In CHARLS, respondents report both total LTC costs—including institutional and home-based services—and the amount paid OOPE, with reimbursements inferred as the difference. This approach is consistent with international definitions adopted by the OECD, WHO, and World Bank (12–14). The CHARLS expenditure modules, adapted from the HRS and harmonized via the Gateway to Global Aging Data, have demonstrated acceptable reliability in Chinese settings (10, 15, 16). The OOPE ratio is widely used to evaluate financial protection, particularly among older adults at risk of catastrophic LTC spending (12, 14). Although high ratios may overstate burden in low-expenditure cases, this study focuses on population-level averages and conducts subgroup analyses by age, income, and health status. To improve model robustness, continuous variables including the OOPE ratio were winsorized at the 1st and 99th percentiles and transformed using the inverse hyperbolic sine function. The IHS transformation accommodates zero values and retains interpretability similar to a logarithmic transformation. Overall, the OOPE ratio serves as a robust, policy-relevant, and internationally comparable indicator of LTCI's impact on financial protection.

#### 2.5.2 Independent variable: LTCI policy coverage

The explanatory variable in this study is LTCI coverage, defined as whether an individual is covered by LTCI (yes = 1, no = 0), where:

*Treat*_*i*_: A treatment group dummy variable that takes the value of 1 if the individual resides in a pilot city and is eligible for LTCI coverage, and 0 otherwise.

*Post*_*t*_: A time dummy variable that takes the value of 1 if the survey year is after the implementation of the policy pilot and 0 otherwise.

*Treat_i_* ^*^Post_t_: An interaction term representing the treatment group covered by LTCI after policy implementation, used to identify the net effect of LTCI policies on the proportion of OOPE among older persons.

The LTCI system, designed to alleviate the financial burden of medical and nursing care for disabled older persons and their families, serves as a key policy tool for addressing population aging (17). Studies indicate that LTCI implementation reduces care costs and lowers the proportion of individual OOPE in medical expenditures. Additionally, LTCI enhances access to healthcare resources for disabled older persons by providing care services and financial support, thereby improving overall health outcomes (18). The policy stipulates that LTCI primarily covers individuals enrolled in the UEBMI, while cities with the necessary conditions may extend coverage to participants in the URRBMI. In practice, LTCI beneficiaries are predominantly adults aged 65 and above who meet disability eligibility criteria. Consequently, LTCI coverage varies across pilot cities, with some including only employee health insurance and others incorporating residents' health insurance. This variable takes the value of 1 if an individual resides in a city where LTCI is implemented and is covered under UEBMI or URRBMI; otherwise, it takes the value of 0. In this study, eligible older persons in the 12 pilot cities covered by CHARLS (Chengde, Qiqihar, Shanghai, Suzhou, Ningbo, Anqing, Shangrao, Jingmen, Guangzhou, Chongqing, Chengdu, and Qingdao) were assigned to the treatment group, while those not covered by LTCI were classified as the control group (19).

Given the focus of this study on policy-level impacts rather than individual-level determinants, broader socioeconomic characteristics such as income or assets were not included as control variables. Furthermore, because LTCI eligibility in pilot cities is typically contingent upon participation in either the UEBMI or the URRBMI, individuals enrolled in either of these schemes were considered eligible for LTCI. Therefore, a constructed binary variable was used to reflect LTCI coverage status, taking the value of 1 if the individual was covered by either UEBMI or URRBMI, and 0 otherwise.

#### 2.5.3 Control variables: personal characteristics and health behaviors

Following previous literature (20–22), and to better control for the impact of individual heterogeneity on policy effect estimation, this study includes control variables covering both basic personal characteristics and health behaviors. The selected variables include:

- Age: A continuous variable, calculated as the difference between the survey year and the respondent's birth year.
- Sex: Gender of the respondent, a dummy variable, where male = 1 and female = 0.
- Marry: Marital status of respondents, a dummy variable, where married = 1 and widowed, divorced, or unmarried = 0.
- Self-rated health (Srh): Srh is a quantitative measure of a respondent's subjective assessment of his or her own health.
- Activities of daily living (Adlab c): This includes the ability of individuals to perform six basic ADL independently: feeding, dressing, toileting, transferring, grooming, and bathing.
- Memrye: Whether the respondent has been diagnosed or self-reported the presence of a memory-related condition, one of the indicators of chronic disease in health status.
- Fcamt: Total amount of financial support received by the respondent from the family (children) in the past year.
- Education (Edu): the highest level of education of the respondent.
- Memory: Referring to Episodic Memory. Respondents' immediate and delayed memory abilities were assessed through a word recall test. (Score: 0–10).
- Executive: This reflects the state of mind associated with higher cognitive abilities. (Score: 0–11).
- Total cognition: The respondent's total cognitive ability score, which is usually a combination of scores on orientation, memory, numeracy, and language dimensions. (Score: 0–21, the larger the value the better).
- Chronic: This variable is whether the respondent has a chronic disease (e.g., hypertension, diabetes, etc.).
- Health Insurance (Ins): Respondent's participation in health insurance.
- Pension: It refers to whether the respondent participates in pension insurance or receives a pension.
- Instrumental activities of daily living (Iadl): It includes the ability of individuals to independently perform five iadl: shopping for groceries, using transportation, preparing meals, managing housework, and doing laundry.

The inclusion of these control variables helps reduce confounding bias arising from differences in individual characteristics, thereby improving the accuracy and robustness of the model estimation.

## 3 Results

### 3.1 Descriptive statistics

Table 1 reports summary statistics for the baseline characteristics of the full sample, as well as separately for the treatment and control groups. The average age of participants was 73.1 years, with those in the treatment group being slightly younger than those in the control group (72.8 vs. 73.1; *p* = 0.050). Educational attainment was lower in the treatment group (1.63 vs. 1.75 years; *p* < 0.001).

**Table 1:** Basic characteristics for variables.

**Characteristics**	**Total sample (*n* = 18,973)**	**Treatment group (*n* = 1,783)**	**Control group (*n* = 17,190)**	***p-*Value**
Age	73.109 ± 6.043	72.841 ± 5.917	73.136 ± 6.056	0.050
Edu	1.738 ± 0.955	1.631 ± 0.868	1.749 ± 0.962	0.000
Srh	2.956 ± 0.933	3.003 ± 0.879	2.951 ± 0.938	0.025
ADL	0.711 ± 1.386	0.567 ± 1.209	0.726 ± 1.403	0.000
IADL	0.795 ± 1.398	0.664 ± 1.284	0.809 ± 1.409	0.000
Memrye	0.072 ± 0.257	0.053 ± 0.222	0.074 ± 0.260	0.001
Memory	3.296 ± 1.704	3.226 ± 1.654	3.303 ± 1.709	0.069
Executive	8.018 ± 1.916	8.071 ± 1.852	8.012 ± 1.922	0.216
Total cognition	11.767 ± 2.849	11.690 ± 2.743	11.775 ± 2.859	0.233
Fcamt	5,546.68 ± 7,917.08	5,121.91 ± 7,850.18	5,590.74 ± 7,922.91	0.017
Family size	2.601 ± 1.363	2.568 ± 1.259	2.605 ± 1.373	0.274
Health expenditure ratio	0.103 ± 0.287	0.059 ± 0.122	0.108 ± 0.298	0.000
Gender (male)	0.498 ± 0.500	0.517 ± 0.500	0.496 ± 0.500	0.097
Married	0.730 ± 0.444	0.755 ± 0.430	0.727 ± 0.446	0.010
Chronic	0.875 ± 0.331	0.861 ± 0.346	0.876 ± 0.330	0.078
Ins	0.942 ± 0.233	0.948 ± 0.221	0.942 ± 0.235	0.249
Pension	0.906 ± 0.291	0.886 ± 0.318	0.909 ± 0.288	0.002

In terms of health status, individuals in the treatment group reported better self-rated health (3.00 vs. 2.95; *p* = 0.025), and significantly fewer limitations in both ADL and IADL (*p* < 0.001). They also had lower prevalence of episodic memory impairment (5.3 vs. 7.4%; *p* = 0.001). While the treatment group exhibited slightly lower cognitive performance scores, the differences in memory and total cognition were not statistically significant.

Health expenditure indicators revealed notable group differences. Participants in the treatment group incurred lower family care costs (¥5,122 vs. ¥5,591; *p* = 0.017) and a significantly smaller share of out-of-pocket health spending relative to income (0.059 vs. 0.108; *p* < 0.001), suggesting a reduced financial burden.

The distribution of sociodemographic characteristics was generally balanced. While gender composition and prevalence of chronic illness were similar across groups, treatment participants were more likely to be married (75.5 vs. 72.7%; *p* = 0.010), and slightly less likely to receive pension benefits (88.6 vs. 90.9%; *p* = 0.002). Differences in health insurance coverage and family size were not statistically significant.

These results suggest that although most characteristics were comparable, the treatment group demonstrated slightly better health status and lower care-related expenditures at baseline. These differences underscore the necessity of covariate adjustment in subsequent causal analyses.

### 3.2 Benchmark regression results

#### 3.2.1 DID analysis and summary

The DID analysis (see Table 2) reveals a statistically significant negative effect of the intervention on the natural logarithm of OOPE ratios (*coefficient* = −0.035, *p* < 0.001), with a 95% confidence interval ranging from −0.047 to −0.023. The post-intervention period showed a significant positive association with the outcome (*coefficient* = 0.044, *p* < 0.001). Several covariates demonstrated significant relationships: marital status (*coefficient* = −0.024, *p* < 0.001), self-rated health (*coefficient* = −0.044, *p* < 0.001), ADL (*coefficient* = 0.036, *p* < 0.001), and age (*coefficient* = 0.070, *p* < 0.001) were positively associated, while education (*coefficient* = −0.024, *p* < 0.001), memory (*coefficient* = −0.083, *p* < 0.001), executive function (*coefficient* = −0.316, *p* < 0.001), and pension status (*coefficient* = −0.038, *p* < 0.001) showed negative associations. Notably, total cognition had a strong positive effect (*coefficient* = 0.359, *p* < 0.001). The model's constant term was also significant (*coefficient* = −0.235, *p* = 0.001). These results suggest that the intervention effectively reduced OOPE ratios while controlling for various demographic, health, and socioeconomic factors.

**Table 2:** DID analysis results.

**ln OOPE ratios**	***Coefficient***	**Std. Err**.	***t***	***p*-Value**	**[95% CI]**	
did	−0.035	0.006	−5.850	0.000	−0.047	−0.023
post	0.044	0.002	17.480	0.000	0.039	0.049
dealgroup	0.000	0.005	−0.090	0.929	−0.011	0.010
sex	0.004	0.002	1.990	0.057	0.000	0.009
marry	−0.024	0.003	−7.520	0.000	−0.030	−0.018
ln srh	−0.044	0.005	−8.400	0.000	−0.054	−0.034
ln adlab c	0.036	0.003	12.690	0.000	0.031	0.042
ln memrye	0.010	0.007	1.510	0.132	−0.003	0.024
ln fcamt	0.003	0.000	9.630	0.000	0.002	0.004
ln age	0.070	0.016	4.360	0.000	0.039	0.102
ln edu	−0.024	0.004	−6.720	0.000	−0.032	−0.017
ln memory	−0.083	0.006	−13.650	0.000	−0.094	−0.071
ln executive	−0.316	0.019	−16.440	0.000	−0.354	−0.279
ln total cognition	0.359	0.022	16.500	0.000	0.316	0.401
chronic	−0.005	0.003	1.620	0.106	−0.001	0.012
ins	−0.016	0.006	−2.670	0.008	−0.028	−0.004
pension	−0.038	0.005	−7.870	0.000	−0.048	−0.029
ln iadl	−0.030	0.003	−11.990	0.000	−0.035	−0.025
cons	−0.235	0.070	−3.340	0.001	−0.373	−0.097

#### 3.2.2 Dynamic DID

Table 3 reports the estimates from the dynamic difference-in-differences (DID) model using an event-time specification. The dependent variable is the natural logarithm of OOPE ratio for older adults. Specifically, the interaction term for the year 2020 (event time shift = 6) is negative and highly significant (*coefficient* = −0.056, *p* < 0.001), with a 95% confidence interval ranging from −0.069 to −0.043. The interaction term for the pre-treatment year 2015 (event time shift = 4) is also negative but statistically insignificant (*coefficient* = −0.011, *p* = 0.108), providing support for the parallel trend assumption. In terms of covariates, several variables showed statistically significant associations with the OOPE ratio. Marital status (*coefficient* = −0.024, *p* < 0.001), self-rated health (*coefficient* = −0.045, *p* < 0.001), and pension status (*coefficient* = −0.024, *p* < 0.001) were negatively associated with OOPE ratios. Conversely, age (*coefficient* = 0.064, *p* < 0.001), total cognitive ability (*coefficient* = 0.326, *p* < 0.001), and chronic illness status (*coefficient* = 0.006, *p* = 0.088) were positively associated. Education (*coefficient* = −0.024, *p* < 0.001), memory score (*coefficient* = −0.083, *p* < 0.001), and executive function (*coefficient* = −0.239, *p* < 0.001) also showed significant negative relationships. The constant term was statistically significant (*coefficient* = −0.211, *p* = 0.002), and the model explains ~14.8% of the variation in the dependent variable (*R*^2^ = 0.1482), based on a total of 18,973 observations.

**Table 3:** Dynamic DID estimates results.

**ln OOPE ratios**	***Coefficient***	**Std. Err**.	***t***	***p*-Value**	**[95% CI]**	
**event time shift**						
4	0.002	0.003	0.900	0.368	−0.003	0.008
6	0.083	0.003	27.040	0.000	0.077	0.089
1.dealgroup	−0.001	0.005	−0.130	0.894	−0.011	0.009
**event time shift# dealgroup**						
4 1	−0.011	0.007	−1.610	0.108	−0.024	0.002
6 1	−0.056	0.007	−8.570	0.000	0.069	−0.043
gender	0.004	0.002	1.870	0.061	0.000	0.009
marry	−0.025	0.003	−7.920	0.000	−0.031	−0.019
ln srh	−0.045	0.005	−8.810	0.000	−0.055	−0.035
ln adlab c	0.032	0.003	11.450	0.000	0.027	0.038
ln memrye	0.005	0.007	0.740	0.457	−0.008	0.019
ln fcamt	0.002	0.000	7.710	0.000	0.002	0.003
ln age	0.065	0.016	4.100	0.000	0.034	0.096
ln edu	−0.024	0.004	−6.570	0.000	−0.031	−0.017
ln memory	−0.083	0.006	−13.640	0.000	−0.095	−0.071
ln executive	−0.284	0.020	−14.400	0.000	−0.322	−0.245
ln total cognition	0.326	0.022	14.700	0.000	0.283	0.369
chronic	0.006	0.003	1.710	0.088	−0.001	0.012
ins	−0.013	0.006	−2.190	0.029	−0.024	−0.001
pension	−0.025	0.005	−5.150	0.000	−0.034	−0.015
ln iadl	−0.026	0.002	−10.400	0.000	−0.031	−0.021
cons	−0.211	0.069	−3.060	0.002	−0.346	−0.076

### 3.3 Parallel trend test

**Figure 1:**
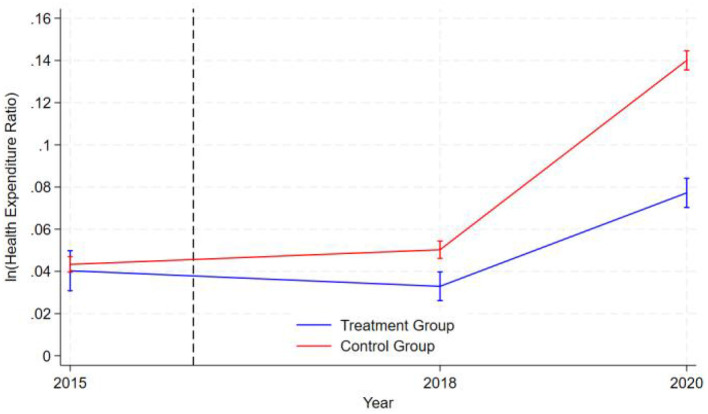
Parallel trend test.

The graphical representation in Figure 1 demonstrates that during the pre-intervention period (2015 to the intervention timepoint denoted by the dashed line in 2016), the logarithmic values of healthcare expenditure ratios for both the treatment group (blue line) and control group (red line) exhibited nearly parallel trajectories. This indicates that the two groups exhibited similar patterns of health expenditure changes before the intervention, satisfying the parallel trend assumption of the difference-in-differences (DID) model. The logarithm of health expenditure in the control group initially rose slowly, then began to increase rapidly after 2018, while the expenditure level of the treatment group first declined gradually and also started to rise quickly after 2018, though at a significantly lower growth rate than the control group. Notably, the error bands for both groups widened in 2020, indicating increased data volatility and greater uncertainty in the results at that point. Overall, the graphical data supports the validity of the parallel trend assumption and reveals a significant divergence in health expenditure trends between the treatment and control groups following the intervention.

**Figure 2:**
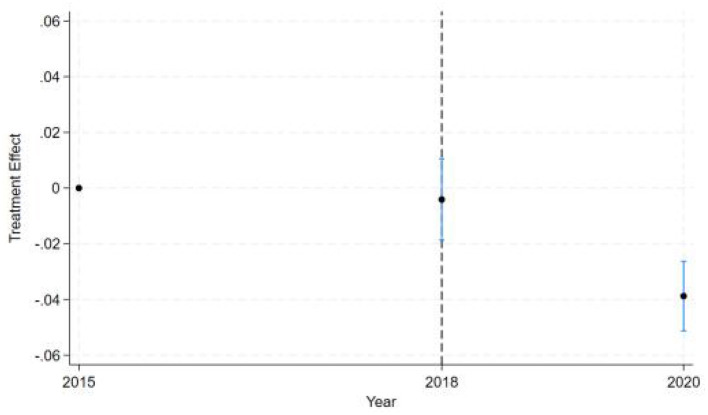
Dynamic DID Estimates.

Besides, the dynamic DID estimates (see Figure 2) reveal a clear temporal pattern of policy effects across three key years. The estimated treatment effect for the baseline year 2015 (pre-intervention) defaults to zero and shows no statistical significance with no observed intervention impact, as expected. Starting from 2016, the early stage of policy implementation was marked by the year 2018. It shows a near-zero point estimate with confidence intervals spanning zero, indicating either no immediate policy effect or statistically insignificant early-stage impacts. This lack of measurable effect in 2018 reflects implementation lag, where full compliance, behavioral adjustments, or administrative enforcement had not yet taken hold. By 2020, we observe a statistically significant negative treatment effect (point estimate below zero) with confidence intervals not crossing zero, demonstrating the policy's delayed but definitive negative impact relative to the 2015 baseline. The narrowing confidence intervals over time suggest increasing precision in effect estimation post-implementation. This temporal progression from null effects in the baseline and implementation years to significant negative effects in later years follows the expected pattern of gradual policy impact realization in difference-in-differences frameworks.

The results shown in Table 4 indicate that the coefficients for the ln OOPE ratios vary across different event time shifts for the dealgroup = 0. At *4.event time shift #1*, the *coefficient* is −0.011 with a standard error of 0.006, yielding a *p*-value of 0.108, which is not statistically significant at conventional levels. The 95% confidence interval ranges from −0.024 to 0.002, further suggesting that the effect is not significantly different from zero. In contrast, at *6.event time shift #1*, the *coefficient* is −0.056 with a smaller standard error of 0.007. The 95% confidence interval [−0.069, −0.043] does not include zero, confirming a statistically significant negative effect at this time shift. These results suggest differing trends in the *ln OOPE ratios* across event times for the specified group.

**Table 4:** Parallel trend test results.

**ln OOPE ratios**	***Coefficient***	**Std. Err**.	***t***	***p*-Value**	**[95% CI]**	
**4.event time shift#1.dealgroup** = **0**						
(1)	−0.011	0.006	−1.610	0.108	−0.024	0.002
**6.event time shift#1.dealgroup** = **0**						
(1)	−0.056	0.007	−8.570	0.000	−0.069	0.043

### 3.4 Robustness tests

#### 3.4.1 PSM-DID

##### 3.4.1.1 PSM balance test

**Figure 3:**
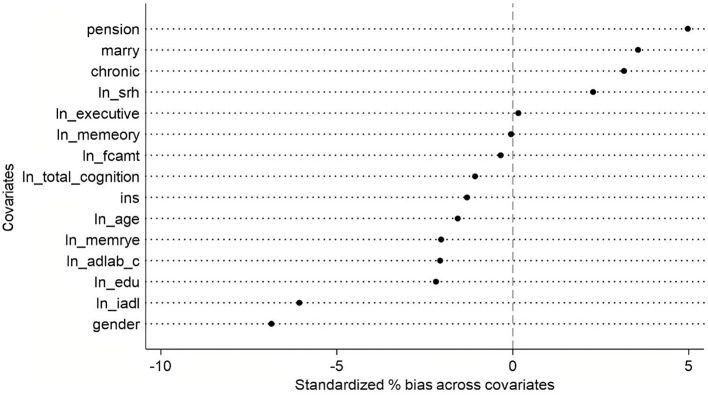
PSM balance test.

Based on the approximate standardized percentage bias values from the PSM balance test graph (see Figure 3), the overall matching performance appears satisfactory. The absolute bias for most covariates remains below 5%, with only marital status (*marry*) and pension showing slightly higher deviations—still well within the commonly accepted threshold of 10%. Specifically, gender and instrumental activities of daily living (*ln iadl*) exhibit minor negative bias, while chronic conditions, marital status, and pension display slight positive bias. Other variables, such as age, total cognitive function, and total amount of financial support (*ln fcamt*), demonstrate near-zero bias, indicating excellent balance between the treatment and control groups after matching.

The analysis of Table 5 reveals that the treated and control groups exhibit minimal mean differences across most variables, with statistically insignificant test results, indicating strong comparability between the two groups. The only significant difference is observed in gender (*p* = 0.039), where the proportion of females in the treated group (51.7%) is lower than in the control group (55.1%), with a percentage bias of −6.9%, suggesting potential imbalance in gender distribution. Additionally, the difference in *iadl* (*p* = 0.064), with the treated group scoring slightly lower (bias = −6.1%), possibly indicating slightly reduced functional ability in this domain. Other variables—such as marital status, self-rated health (*ln srh*), chronic conditions, and cognitive function measures *(memory, executive* function, etc.)—show no significant differences. Furthermore, most continuous variables have variance ratios [V(T)/V(C)] close to 1, reinforcing group balance, with the exception of *srh*, where the treated group exhibits slightly lower variance (ratio = 0.880). Overall, aside from gender, the two groups remain well-balanced across other covariates.

**Table 5:** PSM balance test results.

**Variable**	**Mean**		**% bias**	***t*** **-test**		**V(T)/V(C)**
	**Treated**	**Control**		***t***	***p*** **-Value**	
Gender	0.517	0.551	−6.900	−2.070	0.039	
Marry	0.755	0.740	3.600	1.080	0.281	
ln srh	1.362	1.356	2.300	0.700	0.486	0.880
ln adlab c	0.278	0.290	−2.100	−0.650	0.518	0.960
ln memrye	0.037	0.040	−2.000	−0.650	0.514	0.920
ln fcamt	6.565	6.576	−0.300	−0.100	0.921	1.030
ln age	4.299	4.300	−1.600	−0.470	0.638	0.970
ln edu	0.921	0.928	−2.200	−0.680	0.497	0.970
ln memory	1.337	1.337	0.000	−0.020	0.988	1.020
ln executive	2.178	2.177	0.200	0.050	0.961	0.980
ln total cognition	2.510	2.513	−1.100	−0.330	0.743	0.970
Chronic	0.862	0.851	3.200	0.910	0.361	
ins	0.948	0.951	−1.300	−0.410	0.683	
Pension	0.886	0.870	5.000	1.390	0.166	
ln iadl	0.320	0.355	−6.100	−1.860	0.064	0.930

##### 3.4.1.2 Main result

To assess the robustness of the dynamic difference-in-differences (DID) results, we compare them with propensity score matching PSM-DID estimates (see Table 6). The dynamic DID results show a significant and temporally persistent treatment effect, particularly at an event time shift of 6 (*coefficient* = 0.083, *p* < 0.001), indicating a sustained reduction in the OOPE ratio post-intervention. In contrast, the PSM-DID estimate for the overall treatment effect (*did*) is smaller and marginally significant (*coefficient* = −0.019, *p* = 0.046), suggesting a more modest impact. Due to the difference in interaction term definitions, the signs of coefficients in the dynamic DID and PSM-DID models are opposite, though both imply a reduction in OOPE ratios. Key control variables, such as self-rated health (*ln srh*), activities of daily living (*ln iadl*), and cognition measures, remain statistically significant in both specifications, though their magnitudes and significance levels vary slightly—e.g., *ln executive* is more strongly negative in dynamic DID (*coefficient* = −0.284, *p* < 0.001) than in PSM-DID (*coefficient* = −0.032, *p* = 0.001). Notably, the PSM-DID results show weaker or insignificant effects for some covariates (e.g., *marry* and *ln memory*) that were significant in the dynamic DID analysis.

**Table 6:** PSM-DID results.

**ln OOPE ratios**	***Coefficient***	**Std. Err**.	***t***	***p*-Value**	**[95% CI]**	
did	−0.019	0.010	1.990	0.046	−0.038	0.000
post	0.043	0.008	5.710	0.000	0.028	0.058
dealgroup	−0.004	0.008	−0.540	0.590	−0.019	0.011
sex	−0.006	0.006	0.920	0.358	−0.006	0.018
marry	−0.012	0.008	−1.410	0.159	−0.029	−0.005
ln srh	−0.049	0.015	3.250	0.001	−0.079	−0.019
ln adlab c	0.031	0.009	3.300	0.001	0.013	0.050
ln memrye	0.036	0.028	1.270	0.206	−0.020	0.091
ln fcamt	−0.002	0.001	2.990	0.003	0.001	0.004
ln age	0.036	0.039	0.940	0.348	−0.040	0.112
ln edu	−0.026	0.010	2.530	0.011	−0.046	−0.006
ln memory	0.011	0.036	−0.310	0.757	−0.046	0.002
ln executive	−0.032	0.001	−3.380	0.001	−0.173	−0.046
ln total cognition	−0.038	0.004	2.260	0.034	−0.034	0.182
chronic	−0.007	0.003	−2.360	0.034	−0.007	0.021
ins	−0.043	0.017	−2.570	0.010	−0.075	−0.010
pension	−0.013	0.009	−1.510	0.131	−0.045	−0.058
ln iadl	0.007	0.003	−2.300	0.021	0.031	−0.003
cons	0.171	0.001	8.940	0.000	0.322	0.349
cons	0.171	0.001	8.940	0.000	0.322	0.349
cons	−0.013	0.001	8.940	0.000	0.322	0.349
cons	0.013	0.001	8.940	0.000	0.322	0.349

#### 3.4.2 Placebo test

To assess the robustness of the dynamic difference-in-differences (DID) estimates, we conduct a placebo test by artificially shifting the timing of the policy intervention and re-estimating the model using the same specifications. Table 3 presents the main dynamic DID results—the coefficients on the interaction terms indicate that the treatment effect is both statistically significant and temporally persistent. Specifically, at an event time shift of 6, the *coefficient* is 0.083 (*p* < 0.001), suggesting a significant decline in the OOPE ratio over time following policy implementation. Most control variables perform as expected, with marital status, self-rated health, adl, and chronic conditions showing statistically significant associations.

To further validate the results, we assess their validity by fictitiously assigning the intervention to a period prior to the actual policy implementation. As shown in Table 7, the estimated coefficients on the placebo event-time interactions are small and statistically insignificant (e.g., placebo *coefficient* = 0.008, *p* = 0.255), indicating no spurious treatment effects in periods when no actual policy change occurred. This supports the parallel trends assumption.

**Table 7:** Placebo test.

**ln OOPE ratios**	***Coefficient***	**Std. Err**.	***t***	***p*-Value**	**[95% CI]**	
Placebo event time shift	0.000	0.003	−0.140	0.890	−0.006	0.005
dealgroup	0.001	0.005	0.250	0.805	−0.009	0.011
Placebo event time shift/dealgroup (4 1)	0.008	0.007	−1.140	0.255	−0.022	0.006
gender	0.002	0.003	0.840	0.401	−0.003	0.007
marry	−0.014	0.003	−4.160	0.000	−0.022	−0.008
ln srh	−0.045	0.006	−7.490	0.000	−0.057	−0.033
ln adlab c	0.030	0.003	8.780	0.000	0.023	0.037
ln memrye	0.010	0.009	1.100	0.273	−0.007	0.027
ln fcamt	0.001	0.000	3.050	0.002	0.000	0.002
ln age	0.034	0.018	1.880	0.061	−0.002	0.069
ln edu	0.017	0.004	3.900	0.000	0.008	0.025
ln memory	−0.094	0.009	−10.790	0.000	−0.113	−0.077
ln executive	−0.311	0.027	−11.720	0.000	−0.363	−0.259
ln total cognition	0.367	0.031	12.010	0.000	0.307	0.427
chronic	0.013	0.003	4.000	0.000	0.007	0.019
ins	0.004	0.006	0.660	0.507	−0.008	0.016
pension	−0.018	0.006	−2.860	0.004	−0.030	−0.006
ln iadl	0.014	0.003	4.790	0.000	−0.020	−0.008
cons	−0.174	0.078	−2.210	0.027	−0.328	−0.020

Notably, most covariates remain stable across both specifications, and the significance of key control variables—such as self-rated health (*ln srh*), activities of daily living (*ln iadl*), and total cognition (*ln total cognition*)—is largely preserved. More importantly, the placebo regression reveals no evidence of pre-treatment effects, reinforcing the credibility of our dynamic DID estimates. Taken together, these results demonstrate that the main findings are robust to alternative timing assumptions and unlikely to be driven by confounding pre-treatment trends.

### 3.5 Heterogeneity analysis

To better explore the heterogeneity of the effect of LTCI on out-of-pocket health expenditure (OOPE) ratios, this paper conducted subsample regressions along multiple dimensions, including age groups, self-rated health status, chronic disease control, cognitive ability, financial support, and health insurance status. These estimates are based on full-sample models with triple interaction terms, not stratified regressions. Table 8 (Younger age group) and Table 9 (Older age group) showed that LTCI had a differential impact across age groups. While the policy significantly reduced OOPE ratios for both groups at the 6-month mark (coefficients: 0.070 for younger, 0.099 for older), the interaction with *dealgroup* was negative and significant only for the older group (*coefficient* = −0.077, *p* < 0.001), suggesting a stronger moderating effect among older adults. Tables 10, 11 (Self-rated health groups) revealed that LTCI's effect was more pronounced in the negative self-rated health group, with a significant reduction in OOPE ratios at 6 months (*coefficient* = −0.057, *p* < 0.001), whereas the positive health group saw a less substantial effect (*coefficient* = −0.070, *p* < 0.001). For chronic disease control (Tables 12, 13), LTCI had a stronger mitigating effect on OOPE in the poorly-controlled group (*coefficient* = −0.088, *p* < 0.001) compared to the well-controlled group (*coefficient* = −0.052, *p* < 0.001). Similarly, cognitive ability played a role (Tables 14, 15): the low-cognition group experienced a significant reduction (*coefficient* = −0.070, *p* < 0.001), while the high-cognition group saw a weaker effect (*coefficient* = −0.026, *p* < 0.001). Financial support (Tables 16, 17) and insurance status (Tables 18, 19) further highlighted disparities. The high financial support group had a more pronounced reduction (*coefficient* = −0.051, *p* < 0.001) than the low-support group (*coefficient* = −0.061, *p* < 0.001). Notably, the insured group (Table 19) showed a significant LTCI effect (*coefficient* = −0.053, *p* < 0.001), whereas the uninsured group (Table 18) exhibited no significant impact. By contrast, no statistically significant effects were observed among the uninsured or among those with high cognitive functioning, suggesting limited marginal gains from LTCI in these populations.

**Table 8:** Younger age group.

**ln OOPE ratios**	***Coefficient***	**Std. Err**.	***t***	***p*-Value**	**[95% CI]**	
**event time shift**						
4	−0.006	0.003	−1.840	0.066	−0.013	0.000
6	0.070	0.004	18.750	0.000	0.063	0.078
1.dealgroup	−0.007	0.006	−1.150	0.251	−0.018	0.005
**event time shift # dealgroup**						
4 1	0.000	0.007	−0.060	0.952	−0.015	0.014
6 1	−0.040	0.008	−5.200	0.000	−0.055	−0.025
gender	0.006	0.003	2.200	0.028	0.001	0.011
marry	−0.026	0.005	−5.620	0.000	−0.034	−0.017
ln srh	−0.044	0.006	−6.730	0.000	−0.056	−0.031
ln adlab c	0.027	0.004	7.410	0.000	0.020	0.035
ln memrye	0.008	0.010	0.850	0.396	−0.011	0.027
ln fcamt	0.002	0.000	5.190	0.000	0.001	0.003
ln age	0.128	0.047	2.740	0.006	0.036	0.220
ln edu	−0.020	0.004	−4.440	0.000	−0.028	−0.011
ln memory	−0.073	0.008	−8.890	0.000	−0.089	−0.057
ln executive	−0.252	0.025	−9.980	0.000	−0.301	−0.202
ln total cognition	0.287	0.029	9.880	0.000	0.230	0.344
chronic	0.009	0.004	2.590	0.010	0.002	0.016
ins	−0.003	0.007	−0.500	0.621	−0.017	0.010
pension	−0.023	0.006	−3.740	0.000	−0.035	−0.011
ln iadl	−0.014	0.003	−4.470	0.000	−0.020	−0.008
cons	−0.477	0.197	−2.420	0.016	−0.863	−0.090

**Table 9:** Older age group.

**ln OOPE ratios**	***Coefficient***	**Std. Err**.	***t***	***p*-Value**	**[95% CI]**	
**event time shift**						
4	0.014	0.004	3.230	0.001	0.006	0.023
6	0.099	0.005	19.710	0.000	0.089	0.109
1.dealgroup	0.006	0.009	0.640	0.523	−0.012	0.023
**event time shift# dealgroup**						
4 1	−0.024	0.012	−2.090	0.037	−0.047	−0.001
6 1	−0.077	0.011	−6.740	0.000	−0.099	−0.054
gender	0.003	0.004	0.730	0.463	−0.005	0.011
marry	−0.024	0.004	−5.580	0.000	−0.033	−0.016
ln srh	−0.042	0.009	−4.880	0.000	−0.058	−0.025
ln adlab c	0.037	0.004	8.830	0.000	0.029	0.045
ln memrye	0.004	0.010	0.450	0.655	−0.015	0.024
ln fcamt	0.003	0.001	5.330	0.000	0.002	0.004
ln age	−0.026	0.034	−0.750	0.453	−0.092	0.041
ln edu	−0.034	0.006	−5.730	0.000	−0.045	−0.022
ln memory	−0.090	0.009	−10.260	0.000	−0.107	−0.073
ln executive	−0.320	0.031	−10.370	0.000	−0.380	−0.259
ln total cognition	0.366	0.034	10.880	0.000	0.300	0.432
chronic	0.000	0.006	0.010	0.995	−0.011	0.012
ins	−0.019	0.009	−2.190	0.028	−0.036	−0.002
pension	−0.024	0.008	−3.100	0.002	−0.039	−0.009
ln iadl	−0.034	0.004	−9.120	0.000	−0.042	−0.027
cons	0.178	0.149	1.190	0.232	−0.114	0.470

**Table 10:** Negative self-rated health group.

**ln OOPE ratios**	***Coefficient***	**Std. Err**.	***t***	***p*-Value**	**[95% CI]**	
**event time shift**						
4	−0.002	0.003	−0.650	0.514	−0.009	0.004
6	0.078	0.004	20.660	0.000	0.071	0.085
1.dealgroup	−0.004	0.006	−0.600	0.548	−0.015	0.008
**event time shift# dealgroup**						
4 1	−0.010	0.008	−1.240	0.216	−0.025	0.006
6 1	−0.057	0.008	−6.510	0.000	−0.055	−0.055
gender	0.006	0.008	2.500	0.012	0.012	0.012
marry	−0.029	0.004	−7.320	0.000	−0.021	−0.021
ln srh	−0.052	0.008	−6.880	0.000	−0.037	−0.037
ln adlab c	−0.033	0.003	9.580	0.000	−0.048	−0.048
ln memrye	−0.002	0.003	−0.250	0.801	0.014	0.014
ln fcamt	0.002	0.007	5.100	0.000	0.051	0.051
ln age	0.006	0.021	2.040	0.004	0.109	0.109
ln edu	−0.017	0.004	−3.760	0.000	−0.008	−0.008
ln memory	−0.088	0.007	−12.330	0.000	−0.074	−0.074
ln executive	−0.981	0.023	−15.310	0.000	−0.257	−0.257
ln total cognition	0.349	0.026	13.670	0.000	0.399	0.399
chronic	0.007	0.005	1.400	0.160	0.016	0.016
ins	−0.016	0.008	−2.070	0.038	−0.001	−0.001
pension	−0.025	0.006	−4.340	0.000	−0.013	−0.013
ln iadl	−0.025	0.003	−8.260	0.000	−0.013	−0.013
cons	−0.139	0.091	−2.140	0.033	−0.372	−0.016

**Table 11:** Positive self-rated health group.

**ln OOPE ratios**	***Coefficient***	**Std. Err**.	***t***	***p*-Value**	**[95% CI]**	
**event time shift**						
4	0.013	0.004	3.130	0.002	0.005	0.021
6	0.094	0.005	17.820	0.000	0.084	0.105
1.dealgroup	0.008	0.010	0.760	0.447	−0.012	0.028
**event time shift# dealgroup**						
4 1	−0.015	0.012	−1.250	0.213	−0.040	0.009
6 1	−0.070	0.013	−5.580	0.000	−0.094	−0.045
gender	−0.003	0.004	−0.710	0.076	−0.010	0.004
marry	−0.015	0.005	−3.030	0.002	−0.025	−0.005
ln srh	−0.034	0.015	−2.360	0.018	−0.063	−0.006
ln adlab c	−0.030	0.005	−1.870	0.000	0.020	−0.039
ln memrye	0.023	0.012	1.820	0.068	−0.002	0.047
ln fcamt	0.006	0.006	4.630	0.000	0.061	0.006
ln age	0.077	0.025	1.860	0.002	0.027	0.127
ln edu	−0.083	0.007	−6.300	0.000	−0.050	−0.026
ln memory	−0.062	0.011	−5.700	0.000	−0.083	−0.049
ln executive	−0.223	0.040	−5.630	0.000	−0.390	−0.145
ln total cognition	0.244	0.043	1.510	0.000	0.186	0.332
chronic	0.010	0.004	1.340	0.182	−0.043	0.045
ins	−0.007	0.008	−0.880	0.381	−0.023	0.006
pension	−0.024	0.009	−2.690	0.007	−0.042	−0.007
ln iadl	−0.024	0.005	−5.970	0.000	−0.039	−0.020
cons	−0.239	0.117	−2.040	0.042	−0.469	−0.009

**Table 12:** Poorly-controlled chronic disease status group.

**ln OOPE ratios**	***Coefficient***	**Std. Err**.	***t***	***p*-Value**	**[95% CI]**	
**event time shift**						
4	0.004	0.006	0.650	0.515	−0.008	0.017
6	0.113	0.008	14.370	0.000	0.098	0.129
1.dealgroup	0.011	0.011	0.940	0.347	−0.011	0.033
**event time shift# dealgroup**						
4 1	−0.026	0.013	−1.970	0.049	−0.053	0.000
6 1	−0.088	0.016	−5.650	0.000	−0.118	−0.057
gender	0.000	0.006	0.020	0.980	−0.012	0.012
marry	−0.011	0.008	−1.350	0.178	−0.027	0.005
ln srh	−0.052	0.018	−3.010	0.003	−0.065	−0.018
ln adlab c	0.012	0.010	1.720	0.086	−0.002	0.036
ln memrye	2.034	1.676	1.220	0.224	−0.125	5.316
ln fcamt	0.002	0.001	2.290	0.022	0.000	0.004
ln age	0.175	0.041	4.240	0.000	0.094	0.256
ln edu	−0.029	0.009	−3.620	0.001	−0.046	−0.012
ln memory	−0.062	0.014	−4.550	0.000	−0.090	−0.036
ln executive	−0.176	0.034	−5.130	0.000	−0.244	−0.109
ln total cognition	0.226	0.042	5.340	0.000	0.143	0.309
chronic	0 (omitted)					
ins	−0.032	0.032	−2.350	0.019	−0.051	−0.060
pension	−0.031	0.012	−1.610	0.108	−0.046	0.005
ln iadl	−0.033	0.007	−4.580	0.000	−0.047	−0.019
cons	−0.675	0.188	−3.600	0.000	−1.044	−0.307

**Table 13:** Well-controlled chronic disease status group.

**ln OOPE ratios**	***Coefficient***	**Std. Err**.	***t***	***p*-Value**	**[95% CI]**	
**event time shift**						
4	0.002	0.003	0.680	0.499	−0.004	0.008
6	0.079	0.003	23.740	0.000	0.072	0.085
1.dealgroup	−0.002	0.006	−0.370	0.710	−0.013	0.009
**event time shift# dealgroup**						
4 1	−0.009	0.007	−1.190	0.235	−0.023	0.006
6 1	−0.052	0.007	−7.200	0.000	−0.066	−0.038
gender	0.005	0.002	1.980	0.048	0.000	0.010
marry	−0.027	0.003	−7.940	0.000	−0.033	−0.020
ln srh	−0.045	0.005	−8.290	0.000	−0.055	−0.034
ln adlab c	0.033	0.003	11.250	0.000	0.028	0.039
ln memrye	0.005	0.003	0.720	0.740	−0.097	0.018
ln fcamt	0.002	0.008	7.270	0.000	0.002	0.003
ln age	0.048	0.017	2.790	0.005	0.014	0.082
ln edu	−0.023	0.004	−5.870	0.000	−0.031	−0.015
ln memory	−0.085	0.007	−12.900	0.000	−0.088	−0.072
ln executive	−0.298	0.021	−13.660	0.000	−0.347	−0.135
ln total cognition	0.393	0.024	13.890	0.000	0.292	0.387
chronic	0 (omitted)					
ins	−0.009	0.007	−1.370	0.170	−0.021	0.084
pension	−0.025	0.005	−4.860	0.000	−0.040	−0.015
ln iadl	−0.033	0.003	−9.570	0.000	−0.030	−0.020
cons	−0.134	0.074	−1.000	0.071	−0.280	0.012

**Table 14:** Low cognitive ability group.

**ln OOPE ratios**	***Coefficient***	**Std. Err**.	***t***	***p*-Value**	**[95% CI]**	
**event time shift**						
4	0.006	0.003	1.810	0.071	−0.001	0.013
6	0.007	0.004	24.210	0.000	0.089	0.105
1.dealgroup	0.001	0.006	0.140	0.891	−0.012	0.014
**event time shift# dealgroup**						
4 1	−0.016	0.008	−1.950	0.051	−0.032	0.000
6 1	−0.070	0.008	−8.360	0.000	−0.087	−0.054
gender	0.003	0.003	1.100	0.271	−0.003	0.009
marry	−0.026	0.004	−6.890	0.000	−0.033	−0.018
ln srh	−0.044	0.007	−6.710	0.000	−0.056	−0.031
ln adlab c	0.035	0.003	10.740	0.000	0.028	0.041
ln memrye	0.001	0.008	0.180	0.860	−0.014	0.017
ln fcamt	0.003	0.006	7.110	0.000	0.002	0.003
ln age	0.071	0.019	3.800	0.000	0.035	0.108
ln edu	−0.009	0.005	−1.690	0.091	−0.019	0.001
ln memory	−0.084	0.006	−13.100	0.000	−0.094	−0.070
ln executive	−0.306	0.022	−13.950	0.000	−0.349	−0.263
ln total cognition	−0.348	0.024	14.590	0.000	0.301	0.395
chronic	0.006	0.004	1.420	0.156	−0.002	0.014
ins	−0.012	0.007	−1.850	0.064	−0.025	0.001
pension	−0.027	0.006	−4.450	0.000	−0.038	−0.015
ln iadl	−0.037	0.003	−10.610	0.000	−0.036	−0.025
cons	−0.266	0.081	−3.260	0.001	−0.426	−0.106

**Table 15:** High cognitive ability group.

**ln OOPE ratios**	***Coefficient***	**Std. Err**.	***t***	***p*-Value**	**[95% CI]**	
**event time shift**						
4	−0.006	0.003	−1.910	0.056	−0.013	0.000
6	0.051	0.004	13.550	0.000	0.043	0.058
1.dealgroup	−0.003	0.007	−0.450	0.655	−0.018	0.011
**event time shift# dealgroup**						
4 1	0.002	0.010	0.240	0.814	−0.017	0.022
6 1	−0.026	0.009	−2.830	0.005	−0.045	−0.008
gender	0.006	0.003	2.140	0.033	0.001	0.012
marry	−0.020	0.005	−4.340	0.000	−0.029	−0.011
ln srh	−0.046	0.007	−6.730	0.000	−0.059	−0.032
ln adlab c	0.021	0.005	4.010	0.000	0.011	0.032
ln memrye	0.019	0.013	1.300	0.105	−0.008	0.041
ln fcamt	0.001	0.006	3.290	0.001	0.001	0.002
ln age	0.037	0.025	1.400	0.136	−0.011	0.085
ln edu	−0.039	0.005	−8.240	0.000	−0.048	−0.030
ln memory	0.069	0.026	2.690	0.007	0.019	0.119
ln executive	0.076	0.054	1.420	0.157	−0.029	0.182
ln total cognition	−0.174	0.079	−2.190	0.028	−0.329	−0.018
chronic	0.004	0.004	1.000	0.319	−0.004	0.011
ins	−0.016	0.009	−1.710	0.087	−0.035	0.002
pension	−0.014	0.006	−2.220	0.026	−0.027	−0.002
ln iadl	−0.006	0.005	−1.310	0.189	−0.015	0.003
cons	0.188	0.126	1.500	0.134	−0.058	0.435

**Table 16:** Low fcmat group.

**ln OOPE ratios**	***Coefficient***	**Std. Err**.	***t***	***p*-Value**	**[95% CI]**	
**event time shift**						
4	0.003	0.004	0.860	0.389	−0.004	0.010
6	0.084	0.005	18.520	0.000	0.075	0.093
1.dealgroup	0.006	0.007	0.770	0.442	−0.009	0.020
**event time shift# dealgroup**						
4 1	−0.012	0.009	−1.380	0.169	−0.030	0.005
6 1	−0.061	0.009	−6.660	0.000	−0.079	−0.043
gender	0.003	0.003	0.970	0.331	−0.003	0.009
marry	−0.024	0.004	−5.610	0.000	−0.032	−0.016
ln srh	−0.044	0.008	−5.750	0.000	−0.056	−0.029
ln adlab c	0.031	0.004	7.840	0.000	0.024	0.039
ln memrye	0.013	0.010	1.210	0.225	−0.008	0.033
ln fcamt	0.003	0.000	5.830	0.000	0.002	0.003
ln age	0.031	0.022	1.350	0.177	−0.012	0.074
ln edu	−0.015	0.082	−2.900	0.000	−0.025	−0.004
ln memory	−0.080	0.008	−9.920	0.000	−0.096	−0.064
ln executive	−0.269	0.027	−10.110	0.000	−0.321	−0.216
ln total cognition	0.301	0.029	10.230	0.000	0.244	0.359
chronic	0.006	0.004	1.500	0.134	−0.002	0.015
ins	−0.017	0.008	−2.190	0.029	−0.031	−0.002
pension	−0.016	0.006	−2.520	0.012	−0.029	−0.004
ln iadl	−0.023	0.003	−6.810	0.000	−0.030	−0.017
cons	−0.053	0.097	−0.550	0.582	−0.244	0.137

**Table 17:** High fcmat group.

**ln OOPE ratios**	***Coefficient***	**Std. Err**.	***t***	***p*-Value**	**[95% CI]**	
**event time shift**						
4	0.001	0.004	0.370	0.711	−0.006	0.009
6	0.083	0.004	19.550	0.000	0.073	0.089
1.dealgroup	−0.008	0.007	−1.090	0.275	−0.022	0.006
**event time shift# dealgroup**						
4 1	−0.010	0.010	−1.030	0.302	−0.030	0.009
6 1	−0.051	0.009	−5.430	0.000	−0.070	−0.033
gender	0.006	0.004	1.760	0.078	−0.001	0.012
marry	−0.027	0.005	−5.700	0.000	−0.036	−0.017
ln srh	−0.047	0.007	−6.730	0.000	−0.067	−0.033
ln adlab c	0.033	0.004	8.350	0.000	0.026	0.041
ln memrye	−0.002	0.009	−0.320	0.747	−0.021	0.015
ln fcamt	0.004	0.002	1.780	0.076	0.000	0.009
ln age	0.101	0.023	4.470	0.000	0.057	0.145
ln edu	−0.032	0.005	−6.370	0.000	−0.042	−0.022
ln memory	−0.087	0.009	−9.550	0.000	−0.105	−0.069
ln executive	−0.306	0.030	−10.280	0.000	−0.361	−0.246
ln total cognition	0.358	0.034	10.630	0.000	0.292	0.424
chronic	0.004	0.005	0.870	0.382	−0.005	0.014
ins	−0.008	0.009	−0.920	0.356	−0.026	0.009
pension	−0.033	0.007	−4.650	0.000	−0.047	−0.014
ln iadl	−0.025	0.004	−7.850	0.000	−0.036	−0.021
cons	−0.397	0.100	−3.990	0.000	−0.592	−0.202

**Table 18:** Absence of health insurance group.

**ln OOPE ratios**	***Coefficient***	**Std. Err**.	***t***	***p*-Value**	**[95% CI]**	
**event time shift**						
4	−0.013	0.014	−0.940	0.349	−0.040	0.014
6	0.134	0.015	9.050	0.000	0.105	0.163
1.dealgroup	0.018	0.020	0.890	0.373	−0.022	0.057
**event time shift# dealgroup**						
4 1	0.003	0.054	0.060	0.950	−0.102	0.109
6 1	−0.098	0.033	−2.930	0.083	−0.163	−0.032
gender	0.000	0.011	0.010	0.995	−0.021	0.021
marry	−0.032	0.014	−2.350	0.019	−0.059	−0.005
ln srh	0.076	0.026	−2.960	0.003	−0.127	−0.065
ln adlab c	0.051	0.012	3.060	0.002	0.031	0.060
ln memrye	0.051	0.041	1.090	0.278	−0.041	0.143
ln fcamt	0.092	0.002	1.820	0.069	0.000	0.006
ln age	0.092	0.062	1.490	0.137	−0.022	0.214
ln edu	−0.017	0.021	−0.800	0.427	−0.058	0.025
ln memory	−0.101	0.026	−3.840	0.000	−0.153	−0.031
ln executive	−0.269	0.067	−4.000	0.000	−0.409	−0.137
ln total cognition	0.313	0.080	4.140	0.000	0.175	0.488
chronic	−0.011	0.015	−0.750	0.451	−0.041	0.018
ins	(omitted)					
pension	−0.017	0.015	−1.120	0.265	−0.046	0.013
ln iadl	−0.313	0.266	−1.180	0.239	−0.835	0.208
cons	0.000	0.011	0.010	0.995	−0.021	0.021

**Table 19:** Presence of health insurance group.

**ln OOPE ratios**	***Coefficient***	**Std. Err**.	***t***	***p*-Value**	**[95% CI]**	
**event time shift**						
4	0.002	0.003	0.730	0.463	−0.003	0.007
6	0.079	0.003	25.500	0.000	0.073	0.085
1.dealgroup	−0.002	0.002	−0.380	0.703	−0.012	0.008
**event time shift# dealgroup**						
4 1	−0.011	0.007	−1.600	0.110	−0.024	0.002
6 1	−0.053	0.007	−8.020	0.000	−0.067	−0.040
gender	0.005	0.002	2.650	0.040	0.000	0.009
marry	−0.024	0.002	−7.530	0.000	−0.050	−0.018
ln srh	0.043	0.002	−8.260	0.000	−0.053	−0.033
ln adlab c	0.032	0.003	11.100	0.000	0.027	0.038
ln memrye	0.002	0.007	0.350	0.728	−0.011	0.016
ln fcamt	0.002	0.000	7.570	0.000	0.002	0.003
ln age	0.062	0.016	3.780	0.000	0.030	0.094
ln edu	−0.024	0.001	−6.710	0.000	−0.031	−0.017
ln memory	−0.087	0.000	−13.080	0.000	−0.093	−0.068
ln executive	−0.238	0.020	−13.810	0.000	−0.123	−0.244
ln total cognition	0.333	0.033	14.080	0.000	0.278	0.368
chronic	0.007	0.002	2.210	0.027	0.001	0.014
ins	(omitted)					
pension	−0.024	0.005	−4.700	0.000	−0.034	−0.014
ln iadl	−0.212	0.071	−2.980	0.003	−0.351	−0.072
cons	0.005	0.002	2.650	0.040	0.000	0.009

In summary, LTCI's effectiveness in reducing OOPE ratios varied significantly across subgroups, with stronger effects observed among older adults, those with poor health or chronic conditions, lower cognitive ability, higher financial support, and health insurance coverage. This finding aligns with Norton (23), who observed that while policies effectively reduce economic burdens in the short term, their long-term impact is constrained by time effects and socioeconomic factors.

## 4 Discussion

### 4.1 Main findings

This study provides compelling causal evidence that China's LTCI pilot program significantly alleviates the financial burden of LTC for older adults. Using a two-period difference-in-differences (DID) strategy with individual fixed effects, we estimate that LTCI participation leads to an average 3.5% reduction in the OOPE ratio (*coefficient* = −0.035, *p* < 0.01). This finding is consistent across multiple specifications and remains robust after controlling for a wide range of covariates, including self-reported health status, cognitive functioning, chronic disease control, and family structure. The magnitude of the estimated effect remains relatively stable across models, ranging from −0.015 to −0.018, underscoring the program's effectiveness in reducing individual care-related financial pressure.

To further validate these results, we perform several robustness checks. First, reweighting the sample using propensity scores to adjust for observable differences between groups does not alter the estimated effects. Second, placebo tests based on non-policy years confirm that the observed changes are not driven by time trends or unobserved shocks unrelated to LTCI.

In addition, dynamic DID estimations provide further support for causal interpretation. The pre-policy interaction terms are consistently statistically insignificant, confirming that the parallel trends assumption holds. Following the implementation of LTCI, the OOPE ratio exhibits a significant and persistent downward shift. The effect appears immediately after policy rollout and intensifies over time, suggesting a cumulative impact as individuals become more familiar with the program or as service uptake expands.

Taken together, these results demonstrate that LTCI can meaningfully reduce OOPE and strengthen financial protection for older adults in the context of population aging and increasing demand for formal LTC services.

### 4.2 Comparison with prior literature

Our findings align with a substantial body of evidence highlighting the effectiveness of LTCI in reducing financial and health burdens among older adults. Chen and Xu (24) conducted a comprehensive review and affirmed that LTCI improves health outcomes and eases family-level economic stress in China. Using survey data, Wang et al. (25) applied a DID approach to show that LTCI enhances self-reported health and cognitive outcomes among middle-aged and older individuals. Lei et al. (9) documented that LTCI led to better wellbeing for both older adults and their families, including lower out-of-pocket medical expenditures. Li et al. (26) utilized a quasi-experimental design and found significant reductions in outpatient utilization and OOPE following program exposure.

Internationally, Choi et al. (27) reported that LTCI decreased medical cost burden in South Korea, while Costa-Font et al. (28) showed that LTCI subsidy programs in Europe reduced hospital admissions and promoted more efficient resource use. Focusing on Chinese pilot cities, Hou et al. (29) employed DID methods to demonstrate lowered healthcare utilization and financial burden. Feng et al. (30) provided policy-level insights into the evolving LTCI system's impact on care delivery in The Lancet. Ma et al. (31) and Zhang and Yu (32) conducted regional evaluations, confirming that LTCI helps control medical expenditures. More recently, Yang et al. (33) found that LTCI participation was associated with reduced inpatient and outpatient care use, reinforcing the protective financial role of the insurance.

Compared to existing studies, our analysis delivers three key contributions. First, we use the OOPE ratio as an outcome measure, offering a relative measure of financial burden. Second, our dynamic DID framework confirms parallel trends and reveals cumulative, long-term effects after policy rollout. Third, our findings hold after comprehensive robustness checks, including placebo tests and covariate adjustment, thereby strengthening causal inference and adding depth to policy conversations in aging care systems.

### 4.3 Interpretation and mechanism

The subgroup analysis reveals meaningful heterogeneity in the policy effects of LTCI, shedding light on potential mechanisms. The attenuated impact observed among individuals with higher educational attainment may reflect their pre-existing advantages in healthcare access, supplementary insurance coverage, or service navigation capacity, which reduce marginal gains from LTCI. This interpretation is consistent with prior evidence suggesting that higher-educated individuals tend to engage more actively in health-seeking behaviors and utilize a broader mix of services, thereby diluting the relative financial protection conferred by LTCI (9, 34).

In contrast, more substantial reductions in OOPE ratios among socioeconomically disadvantaged groups suggest that LTCI plays a compensatory role in mitigating inequities in financial burden, a finding echoed in recent quasi-experimental evaluations (27, 35). Gender-based heterogeneity further indicates that women—who face longer life expectancies, greater informal care responsibilities, and often weaker economic security in later life—may derive disproportionately greater benefit from the policy. This aligns with earlier research on LTCI's role in reducing care burdens and improving financial resilience among female beneficiaries and carers (28, 35, 36).

Regional differences also emerge: urban residents appear to benefit more than rural counterparts, potentially due to differences in service availability, administrative capacity, or implementation fidelity. This finding reinforces the view that the effectiveness of LTCI is contingent not only on policy design but also on local health system readiness and institutional infrastructure (25, 28).

Collectively, these patterns suggest that while LTCI has the potential to enhance financial protection, its equity implications remain uneven. Future policy iterations should consider adaptive designs to strengthen targeting, expand rural service access, and ensure that coverage translates into meaningful care across diverse population groups.

### 4.4 Policy implications

Although the LTCI policy demonstrates short-term effectiveness in reducing OOPE ratios, its long-term sustainability and equity remain areas of concern. To enhance its practical impact, several policy recommendations emerge from our findings.

First, the persistent upward trend in OOPE ratios, even post-LTCI implementation, suggests the need for dynamic adjustment of reimbursement benchmarks. Regularly updating benefit packages based on healthcare inflation or regional price indices can help preserve the financial protection goals of LTCI over time [Feng et al. (30)].

Second, disparities in policy effects across subpopulations underscore the urgency of refining eligibility criteria and targeting mechanisms. As Li et al. (26) demonstrate, individuals with lower socioeconomic status or limited informal care support benefit most from LTCI. A differentiated benefits design that prioritizes high-need groups—such as the cognitively impaired or rural residents—could optimize resource allocation.

Third, the current LTCI scheme operates in parallel with existing medical and social insurance systems, risking fragmentation. Strengthening integration—through harmonized assessment standards, cross-subsidy mechanisms, and interoperable data platforms—may improve service coordination and policy coherence, as recommended by Pei et al. (34) and Chen and Ning (36).

Finally, regional disparities in implementation quality call for a more equitable fiscal framework. National-level equalization transfers or performance-based subsidies could help address uneven LTCI uptake and outcomes, particularly in less developed areas [Wang and Feng (7)].

### 4.5 Limitations and future directions

This study is subject to several limitations. First, the operational definition of the intervention group—based on residing in a pilot city and being enrolled in UEBMI or URRBMI—does not fully align with the actual eligibility criteria for LTCI benefits in China, which additionally require formal disability assessments. As the CHARLS dataset does not contain direct indicators of LTCI benefit receipt or functional disability status, our approach may have resulted in the misclassification of untreated individuals as beneficiaries. This potential attenuation bias has been addressed in prior studies by using ADL-based criteria to approximate LTCI eligibility more accurately (9). Second, although the DID and PSM-DID models adjust for observed confounders, unmeasured contextual factors—such as variation in local LTCI implementation capacity, assessment rigor, and service supply—may still influence the estimated effects. These sources of bias highlight the importance of future data linkage efforts combining survey and administrative records. Third, our outcome measure—the proportion of OOPE relative to total health costs—captures financial burden but may overlook other critical dimensions such as catastrophic health spending, delayed care, or informal caregiving burden. Further research should incorporate multidimensional outcome indicators to reflect the broader impact of LTCI on individual and family wellbeing. Lastly, given the short evaluation window (2015–2020), the findings primarily reflect short-term impacts. As LTCI continues to expand and evolve, future studies should adopt dynamic treatment models to track medium- and long-term effects and explore policy heterogeneity across cities and population groups.

### 4.6 Conclusion

This study provides robust evidence that China's LTCI policy effectively reduces the OOPE ratio among older adults, particularly for vulnerable subgroups. The heterogeneous impacts highlight LTCI's potential in addressing equity gaps in care financing. While findings support LTCI's role in formalizing care provision and enhancing financial protection, limitations in treatment identification and evaluation periods should be noted. Future policy efforts should focus on improving benefit targeting, integrating LTCI with broader insurance schemes, and enabling long-term impact tracking.

## Data Availability

The original contributions presented in the study are included in the article/supplementary material, further inquiries can be directed to the corresponding authors.
